# Visualizing Catalytic Oxidation of Tryptophan by Nanoceria via an Oligonuclear Cerium Oxo-Complex Model

**DOI:** 10.1021/acs.inorgchem.4c05165

**Published:** 2025-04-10

**Authors:** Sara Targonska, Francesca Greenwell, Tatiana Agback, Gulaim A. Seisenbaeva, Vadim G. Kessler

**Affiliations:** † Department Of Molecular Sciences, Swedish University Of Agricultural Sciences, Box 7015, Uppsala 750 07, Sweden; ‡ Department Of Chemistry, Uppsala University, Box 523, Uppsala 751 20, Sweden

## Abstract

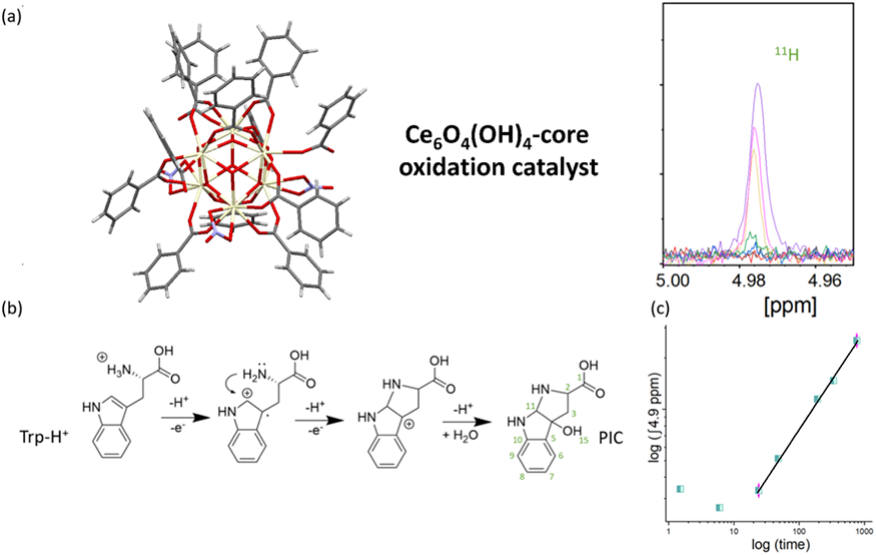

Metal oxide species interact with biologically relevant molecules, which are crucial to the life cycle of plants and animals. Metal oxides can also act as catalysts in various reactions required for proper plant development. In this study, we investigated the hydrolysis of inorganic Ce(IV) precursors in the presence of carboxylic acids, leading to the formation of oligonuclear cerium oxo-complexes. The structure of the species was obtained by X-ray single-crystal studies and found to be hexanuclear, with the composition Ce_6_O_4_(OH)_4_(H_2_O)_2_(NO_3_)_3_(C_7_H_5_O_2_)_9_(C_3_H_7_NO)_4_ (Ce-BA-DMF). The catalytic properties of these complexes on the oxidation of amino acids have been investigated, aiming to establish a transformation mechanism providing insights into both molecular and surface interactions. A redox feature assigned to the CeIV/III couple in the cerium oxo-complex was observed by cyclic voltammetry and found to be sufficiently positive to oxidize tryptophan directly, without the need for intermediate generation of reactive oxygen species. Our findings provide new insights into the possible molecular mechanisms and open the door for more targeted applications of ceria nanoparticles in agriculture and biomedicine.

## Abstract 2

Oxidation of tryptophan by an oligonuclear Ce(IV) complex mimics the nanozyme activity of ceria nanoparticles and proceeds via simultaneous electron-and-proton transfer.

## 1 Introduction

While living organisms primarily consist of organic compounds like proteins, metals are essential for normal cellular function. Metal-containing particles are critical in numerous biological processes as catalysts, cofactors, and regulators in enzymatic and metabolic activities.^1^ Biodedicated nanoparticles and molecules have recently attracted a great deal of attention because of their ability to interact with biomolecules.^2−5^ Numerous studies have been conducted on new compounds with high catalytic abilities toward amino acids, proteins, and DNA analogues. To identify factors that lead to the formation of new structures and determine their function, it is important to structurally characterize the interactions between nanoparticles (NPs) and various molecules in detail.^1,6−10^

NPs based on Ce(IV) are widely used as catalytic agents,^8,11,12^ in electrochemistry,^13^ and wastewater treatment.^14^ Among these applications, oxidative catalysis occupies a very prominent place. While elements commonly found in oxo-complexes enrich these complexes with oxidative properties, providing models for their highest oxidation-state oxides, it would be particularly interesting to isolate species that mimic even more active oxidative catalysts, such as CeO_2_. Closely resembling cerium dioxide, single crystals of cerium oxo-complexes allow a more confident assessment of the relationship between structure and activity.^15,16^

Ce(IV) is a strong oxidative agent (E°CeIV/CeIII vs NHE +1.72 V in 1 M strong acid aqueous solution),^17^ which motivates the study of polyoxometalate NPs made up of Ce(IV). Compared to other rare earth elements, Ce(IV) ions are used in biodedicated materials because of their higher hydrolytic activity, associated with high Lewis acidity. This is related to the +4 oxidation state, high coordination number (up to 12), and relatively fast ligand exchange rate.^7,18^ Despite the fact that Ce(IV)-substituted NPs are widely used as catalysts in different organic transformations, their applications as catalysts for biologically relevant reactions are still relatively unexplored.^19^ However, an earlier study showed that cerium-substituted POM crystals, such as [Ce^IV^(α-PW_11_O_39_)_2_]^10–^, were active as catalytic agents for the selective hydrolysis of the protein—transferrin (Tf). The interaction between Ce-POM and Tf was reported to cause significant changes in the secondary structure, primarily impacting the α-helical content of the protein, but did not involve redox transformations.^20^

Building upon previous work, our group confirmed the involvement of a charge transfer mechanism, supporting theoretical investigations of the interaction between NP and Trp, which provided clear evidence for a new mechanism of direct oxidation.^8^ In this study, we present a new promising cerium oxo-complex oxidation catalyst, together with its complete structural characterization, stability and solubility tests, as well as thermodynamic and kinetic characterization of its activity, proposing a reaction mechanism. The molecular mechanism presented in this paper confirms previously postulated electron and proton transfer pathways in the oxidation of Trp.

## 2 Results and Discussion

To construct an oligonuclear cerium oxo-complex derived from Ce(IV), the synthesis strategy proposed by Gosch et al. was applied,^12^ involving the hydrolysis of cerium ammonium nitrate in the presence of oxidation-resistant organic acids. The synthesis used four different organic substrates: benzoic acid, salicylic acid, acetylsalicylic acid, and *p*-aminobenzoic acid. Two solvent systems were applied for growing crystals from the solution by slow evaporation: water–DMF and water–acetonitrile (here: water–MeCN). Details and conditions of all trials are described in Supporting Information. Among all performed synthesis experiments, only two were successful, including those involving benzoic acid in water–DMF and water–MeCN. The synthesis was successful only in cases where we have solvent as a ligand and it comes into the structure. It is important that the solvent and ligands satisfy the geometric requirements in terms of steric factors as well as the electronic properties of the core, which has a direct impact on synthesis success.^21,22^ In this paper, our attention is focused on the crystals obtained from the water–DMF solution, and details about the crystal structure obtained in the water–MeCN mixture are added in the (Section 2.1).

After 14 days of incubation, a crop of well-defined, yellow cubic/rhombohedral crystals exhibiting smooth faces appeared at the bottom of the vessel. Single X-ray diffraction was applied to investigate the crystals’ composition and determine the crystal structure. Using this method, it was demonstrated that the oxo-complex core is composed of six cerium atoms connected by oxygen atoms. This core was surrounded by nine benzoate, three nitrate, and three DMF ligands bonded to the cerium–oxygen core, and one DMF molecule connected by a hydrogen bond. The crystal structure contains two cerium sites, Ce01 and Ce02. The benzoate ligands are located between two neighboring cerium atoms: Ce01–Ce01, Ce01–Ce02, and Ce02–Ce02. Molecular DMF is bonded to Ce01 atoms (Ce01–O00C = 2.378(6) Å), and nitrate groups are bound to Ce02 atoms (Ce02–O00D = 2.572(0) Å). The hydrogen-bonded DMF molecule is connected to an oxygen atom (2.826 Å), which is bridged between Ce02a and Ce02b. The crystal structure contains three water molecules surrounding each Ce-BA-DMF POM molecule. Two of the water molecules are located between the Ce02 atoms (Ce02a–Ce02c and Ce02b–Ce02c). Hydrogen bonds between these oxygen atoms and water molecules are equal to 2.826 Å. One water molecule is bonded to the oxygen atom located between all three Ce01 atoms, with the hydrogen bond length equal to 2.676 Å. The size of one molecule, as a sum of the covalent radii, is equal to 19.784 Å.

**Figure 1:**
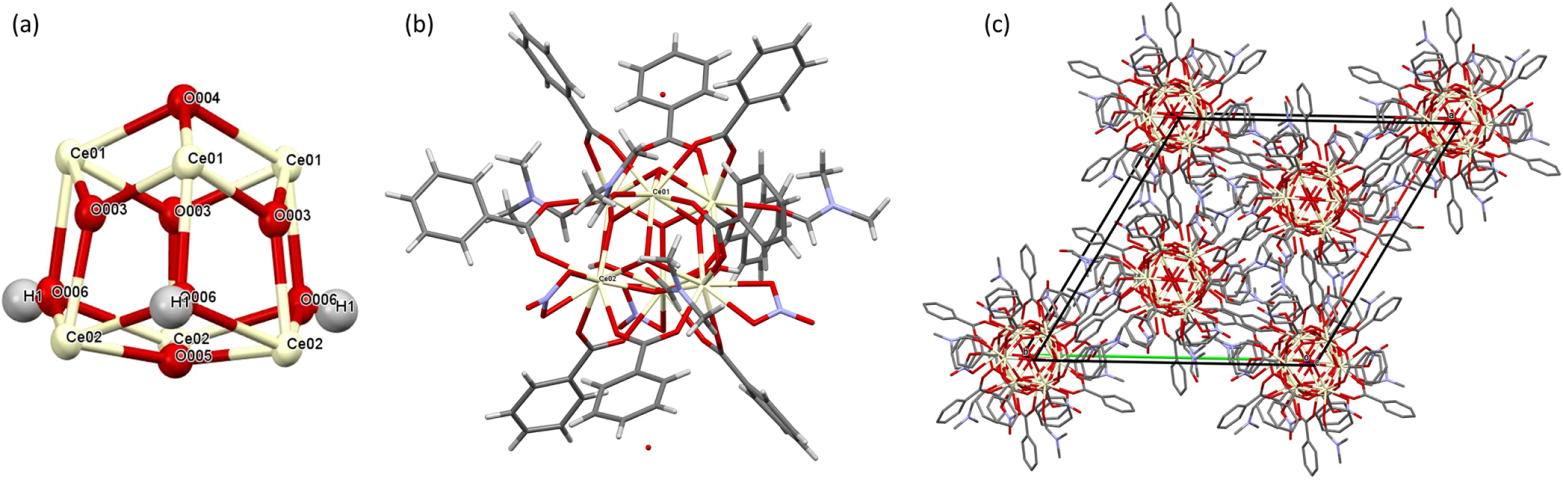
(a) Structure of the Ce_6_O_4_(OH)_4_ core; (b) crystal structure of the Ce-BA-DMF molecule. (c) Packing of THE Ce-BA-DMF unit cell. Color scheme: Ce, green; O, red; N, blue; C, gray.

The chemical composition is Ce_6_O_4_(OH)_4_(H_2_O)_2_(NO_3_)_3_(C_7_H_5_O_2_)_9_(C_3_H_7_NO)_4_, here called Ce-BA-DMF, with a molar mass equal to 2572.52 g/mol, synthesis yield 36%. The obtained composition crystallized in a rhombohedral crystal system belonging to the *R*3̅ space group. It was found that the crystal structure obtained after air drying was stable. The results of refinement for the compound are listed in Table 1. In Figure 1, the ceria-oxygen core, one molecule, and its packing inside the unit cell are shown.

**Table 1:** Details of Unit Cell Parameters and Data Collection of Ce-BA-DMF Crystals

Compound	Ce-BA-DMF
Crystal system	Rhombohedral
Space Group	*R*3̅
Space group number	148
*a*, *b* [Å]	21.8910(7)
*c* [Å]	38.3510(19)
*V* [Å^3^]	15916.2(13)
α, β [°]	90.0
γ [°]	120.0
*T* [K]	273(2)
*Z*	1
*Nr. of obs. independent refl., I > 2σ(I)*	6019
*Residual electron density max*	1.675

### 2.1 Crystal Morphology and Composition

**Figure 2:**
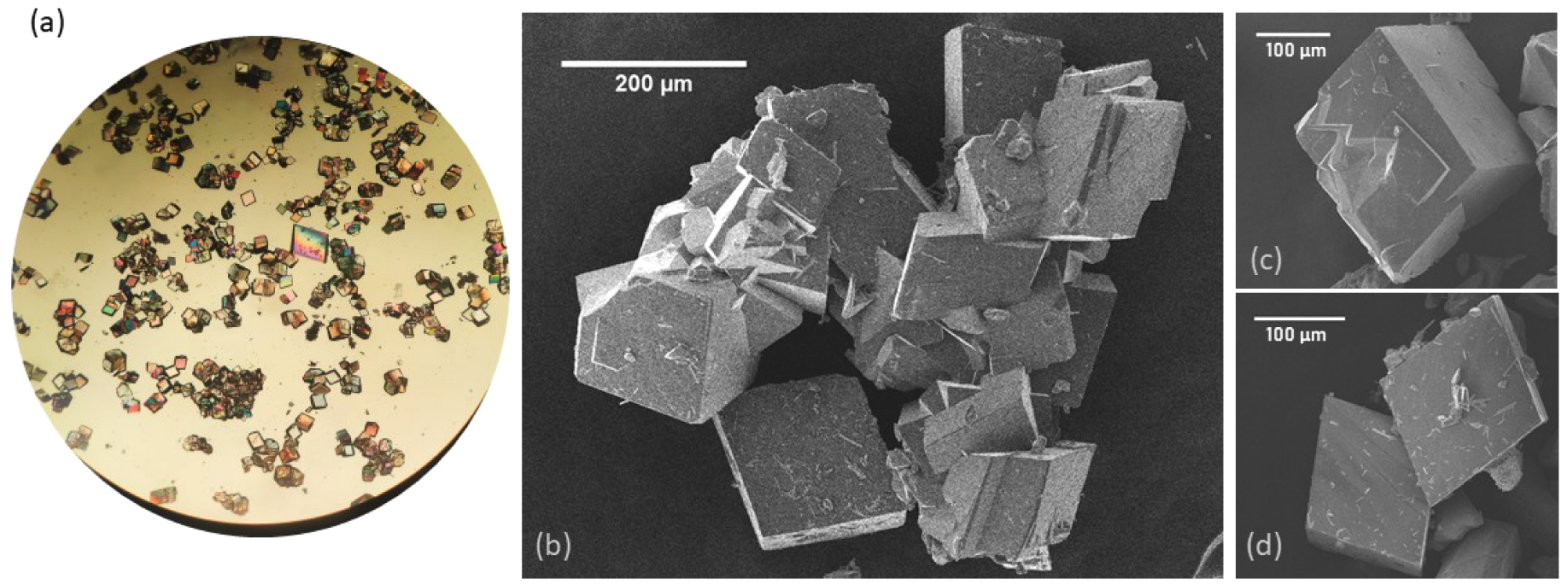
(a) Optical microscope observation and (b–d) SEM images of Ce-BA-DMF crystals.

**Figure 3:**
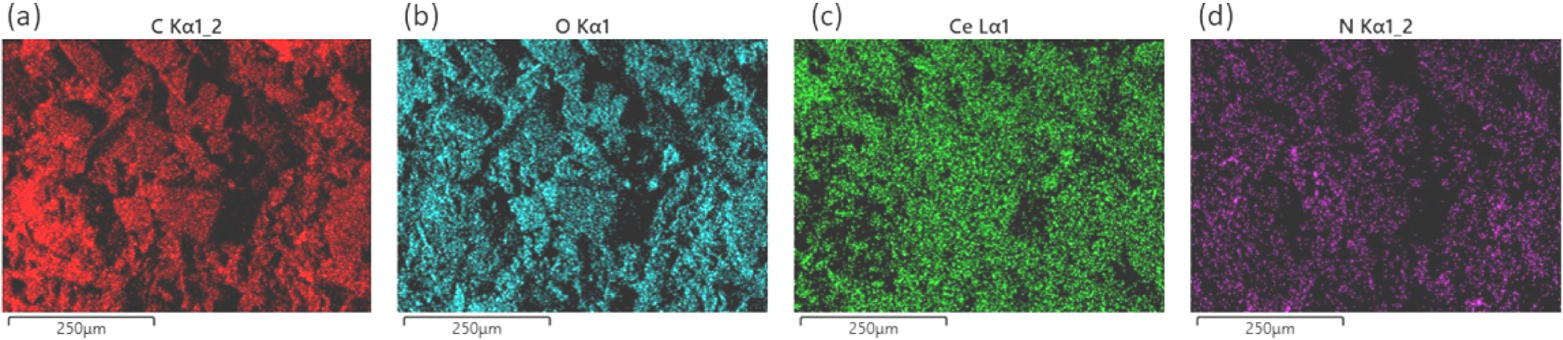
Elemental mapping of the Ce-BA-DMF crystals showing distribution of: (a) carbon, (b) oxygen, (c) cerium, and (d) nitrogen.

Figure 2 presents the scanning electron microscopy (SEM) images of the Ce-BA-DMF crystals. Rhombohedral crystals with lengths in the range of 100–200 μm, colored yellow in the reaction cell, can be observed. The energy-dispersive X-ray spectroscopy (EDS) analysis was conducted to confirm the uniformity of the chemical composition and the presence of cerium atoms. According to the EDS mapping presented in Figure 3, all elements are evenly distributed. The EDS mapping has not revealed any impurities.

### 2.2 pH Stability

**Figure 4:**
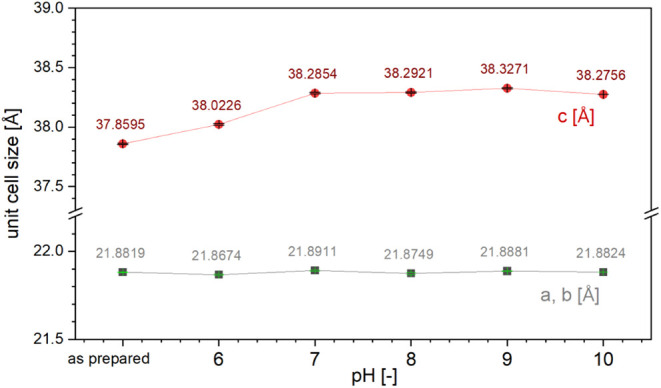
Unit cell parameters after pH treatment.

The material stability was tested in solutions with various pH levels. The HCl solutions at pH 4 and 5, air-equilibrated water with pH 5, and NH_4_OH with pH in the range of 6–10 were investigated in order to study the oxo-complexes’ sensitivity to pH changes. A small amount of Ce-BA-DMF crystals (≈0.01 g) was placed into the solution (5 mL) and stirred for 24 or 48 h. After the given period of incubation, the crystals were removed, placed in oil, and tested by single-crystal X-ray diffraction (XRD) for structure determination. In the case of the samples placed in solutions with pH 6 to pH 10, the single-crystal materials were analyzed after 24 h. The analysis showed that the structure had undergone minor changes, mainly an increase in the length of the *c*-axis from 37.86 to 38.28 Å. Figure 4 illustrates the changes in the size of the unit cells. Despite these changes, the cerium–oxygen core and the organic ligand shells were the same as those of the untreated crystals. Leaving the crystals for 48 h showed that the crystals persisted only in solutions with pH 6 and 7. In solutions with pH 5, the crystal structure was destroyed in less than 24 h. None of the bulk species give enough results to determine the crystal structure.

### 2.3 Solubility at Different pH Levels

To study the solubility of Ce-BA-DMF, 0.01 g of the crystals was placed into 5 mL solutions with varying pH values. After 48 h, the solutions were taken for ICP-OES analysis. The results are displayed in Figure S2. The results show relatively low solubility of Ce-BA-DMF across the whole range of tested pH range (pH 4–10), with higher solubility in acidic media (around 20 mg/L) and very low solubility in basic pH (around 2 mg/L). An unexpectedly high solubility was observed in the case of the pH 6 solution, equal to 104 mg/L.

### 2.4 Cyclic Voltammetry

**Figure 5:**
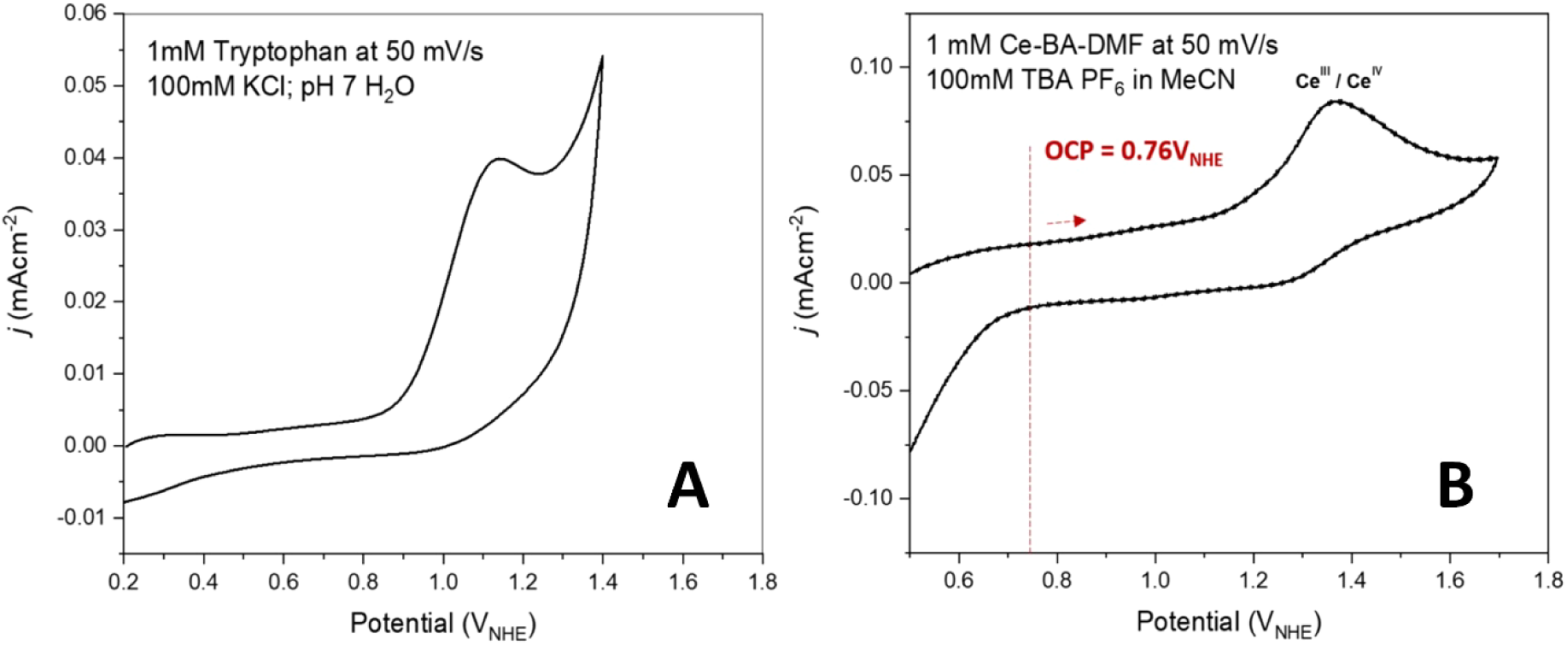
Cyclic voltammograms of (A) tryptophan and (B) Ce-BA-DMF-MeCN.

Electrochemical studies of the behavior of Ce-BA-DMF and tryptophan were investigated using cyclic voltammetry (CV). CVs of 1 mM Ce-BA-DMF were recorded in MeCN using a glassy carbon as the working electrode, where the potential was swept positively from the OCP (Figure 5). CVs were run after the electrode was held at +0.76 V_NHE_ for 10 s before scanning positively from this value. CVs were also run in the opposite direction and appeared exactly the same (see Figure S3b,c). Here, we see an irreversible oxidation peak at 1.33 V vs NHE assigned to the one-electron oxidation of Ce(III) to Ce(IV) ion, due to similar values reported in the literature (Figure 5).^23,24^ While appearing irreversible at low scan rates, suggesting that the Ce(IV) species at the electrode is consumed before the return scan, at faster scan rates, the reduction peak becomes visible and was used to calculate the potential of the Ce(III)/Ce(IV) couple (Figure S3a). Recent reports indicate that only Ce(IV) is present in the hexanuclear cluster,^25^ as well as in the oxo-bridged dimer.^26^ Based on the literature,^25,26^ we attribute the more positive feature to the Ce(IV)/Ce(III) in the CV scan.

To have a meaningful comparison with the literature, CVs of 1 mM tryptophan were measured in an aqueous 1 mM KCl electrolyte. Here, an oxidation peak at +1.14 V vs NHE is observed, which is attributed to the two-electron oxidation of tryptophan, as reported in the literature. While the conditions used are not standardized, the Ce(III)/Ce(IV) couple is more positive than that of tryptophan, suggesting that the Ce(IV) species is oxidative enough to oxidize tryptophan.

### 2.5 Catalysis Kinetics—ABTS

**Figure 6:**
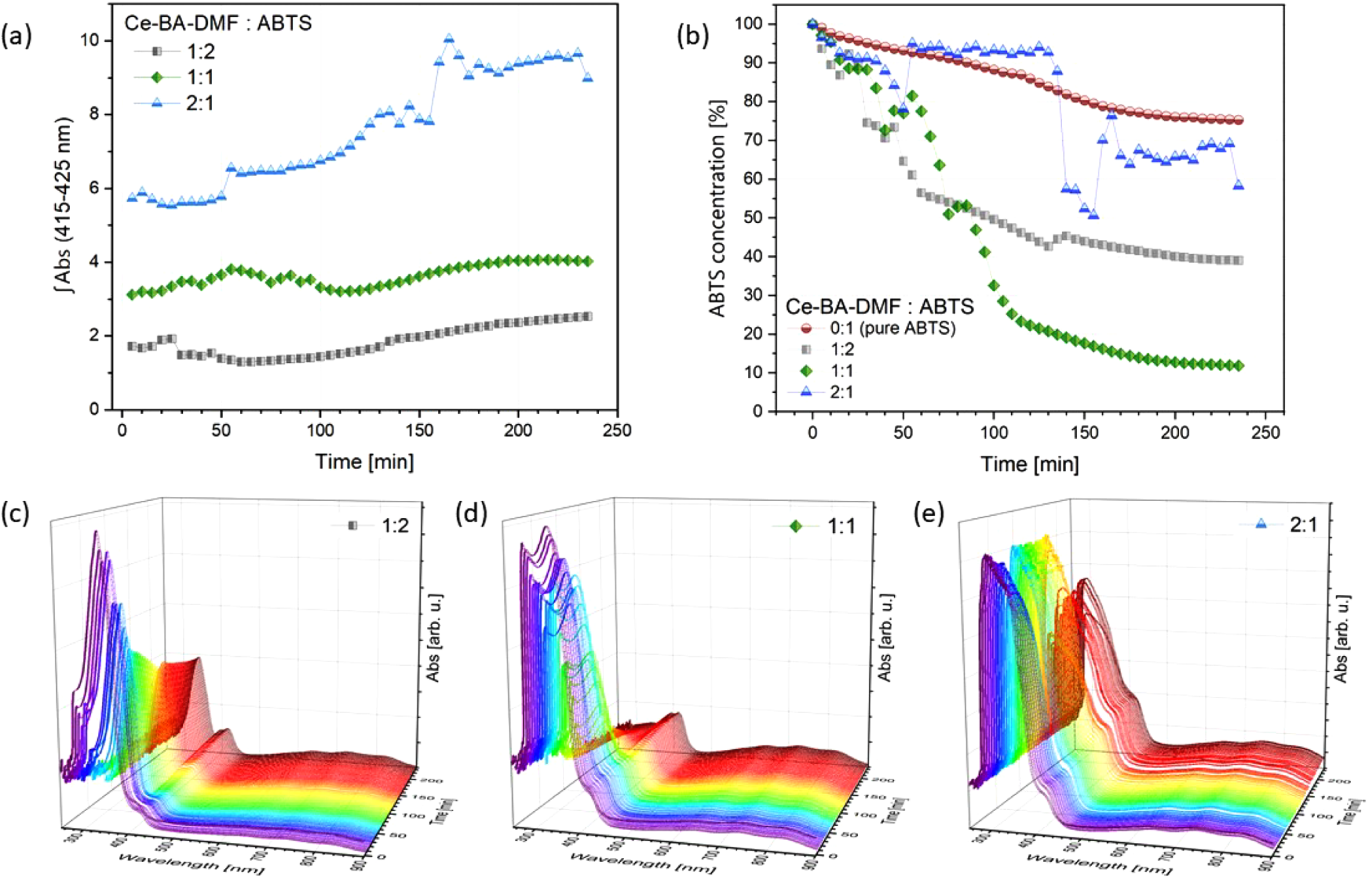
Oxidation process of ABTS in the acetonitrile solution: (a) the mathematical area under UV–vis absorption spectra in the range of 415–425 nm; (b) nonoxidized ABTS concentration; (c) spectra recorded in the ratio of crystals:ABTS 1:2; (d) in the ratio of crystals:ABTS 1:1; (e) in the ratio of crystals:ABTS 2:1.

The ABTS (2,2′-azino-bis(3-ethylbenzothiazoline-6-sulfonic acid) diammonium salt) was used to determine the oxidation characteristics of the Ce-BA-DMF oligonuclear complex. When ABTS is dissolved in water, the oxidation process can be detected by measuring its absorption in the range of blue color wavelengths, with its maximum at 420 nm.^27,28^ As the crystals were shown to be insoluble in water, an additional solvent was added to analyze the compound’s homogeneous reactivity. Therefore, the final tests were done in a mixture of water and acetonitrile or ethanol, respectively. In both tests, the same molar ratios between crystals and ABTS—2:1, 1:1, and 1:2 (crystals:ABTS) were used. A detailed discussion the of water–ethanol solvent system is provided in Section 2.3. The results of the oxidation of ABTS by Ce-BA-DMF in different solvents are presented in Figures 6 and S4.

The kinetics of the cation ABTS^●+^ were derived from concentration changes, monitored as absorption in the range of 415–425 nm in an acetonitrile–water solution, as presented in Figure 6a. The character of oxidation depends on the ratio between Ce(IV) ions and ABTS in the solution. In the case of a higher concentration of ABTS (1:2 Ce-BA-DMF:ABTS), the concentration of the ABTS^●+^ cation increases with some small variations during the first 50 min of the experiment. The oxidation kinetics will be different for solutions with equal molar ratios of Ce(IV) and ABTS, as well as for those with a higher Ce(IV) concentration. If the ratio is equal, there is some variation in the level of the ABTS^●+^ cation in the solution during the100 min from the start of the experiment. With increasing the duration of incubation, the absorption at 420 nm gradually increases. As a result of the addition of excess Ce(IV) ions, ABTS oxidation experienced uneven growth with periods of rapid increase.

Figure 6b illustrates the changes in ABTS concentration, measured as absorption within the 340–350 nm range. A comparison of ABTS concentration is presented for three ratios between the crystals and ABTS, alongside a control experiment involving ABTS in a mixture of water–MeCN. It is noteworthy that the ABTS is spontaneously oxidized in the solution of water-MeCN, even in the absence of oxidants. After 4 h, there was a 25% decrease in the absorption value. The most efficient oxidation, resulting in a decrease of ABTS concentration to 10% of the starting amount, was observed when an equal amount of crystals was tested . After 70 min of the experiment, a 50% reduction was detected. Under conditions of ABTS excess, a 50% reduction was achieved after 90 min. Subsequently, the decrease became very gradual, implying that the amount of oxidizing agents was insufficient to sustain further oxidation. A stable plateau was maintained with an excess of the Ce-BA-DMF complex until 120 min, after which the ABTS concentration decreased rapidly. It then stabilized once more at 65% of its original value after 160 min of incubation.

The water-stable form of ABTS can be oxidized by one electron to the relatively stable cation ABTS^●+^. Further oxidation leads to the less stable dication ABTS^+2^. Oxidation from the cation ABTS^●+^ to the dication ABTS^+2^ form requires a higher redox potential (1.1 V vs NHE) compared to the first oxidation step (0.69 V vs NHE).^28−30^ According to the observed changes in absorption at 420 nm, we conclude that ABTS is oxidized by the Ce-BA-DMF complex in ethanol–water and acetonitrile–water solutions. Based on this data, we proposed that the first product of oxidation, cation ABTS^●+^ can be further oxidized in the presence of excess Ce(IV) ions. Lower concentrations of Ce(IV) ions are fully consumed for the one-electron oxidation of ABTS. At the same time, analysis of the spectra in the range of 300–370 nm suggests the renewal of a small amount of Ce(IV) or ABTS in its reduced form. Cyclic voltammetry measurements have indicated a redox potential of 1.33 V vs NHE of the Ce-BA-DMF complex. It appears that our analysis is in agreement with the first oxidation step in the ABTS process.

### 2.6 Tryptophan Oxidation—NMR Measurements

The presented study investigates the oligonuclear oxo-complex as molecular models of a paper-bag like structure.^9,31^ The particular noncharged molecule-like species are densely packed into a single crystal as a result of external molecular self-assembly forces. Under different conditions and environments, the model of interaction between polyoxometalate molecules or their single crystal surfaces can be studied. This approach was applied to test the interaction and oxidation potential of particular molecules (homogeneous catalysis) and the surfaces of the crystals (heterogeneous catalysis). Since the Ce-BA-DMF crystals are not soluble in water, in the first attempt, the Ce-BA-DMF crystals were placed into a water solution of tryptophan and incubated. The stability of the catalyst in this process was checked by FTIR, which remained unchanged after 1 week in the Trp solution, both in darkness and under constant UV irradiation (see Figure S10). The second attempt was to investigate molecular interaction in DMSO, which is a good solvent for Ce-BA-DMF crystals as well as Trp. The stability of the catalyst was then confirmed by 1H NMR spectroscopy.

Interactions between metal oxide nanoparticles and biomolecules, such as amino acids, are of interest in the view of the oxidative potential of some metal oxides. Our previous study proved that cerium oxide modified by carboxylate ligands results in the formation of a reactive form of cerium(IV) oxide—CeO_2_(−).^8^ As the tested amino acid, we chose tryptophan because of its essential function in the metabolic processes and plant growth.^32^ According to the literature, different oxidation pathways have been proposed depending on the oxidizer. The oxidation of tryptophan can result in the production of various compounds, including hydroxypyrroloindole carboxylic acid, kynurenine, formylkynurenine, and other indole derivatives.^33−35^

**Figure 7:**
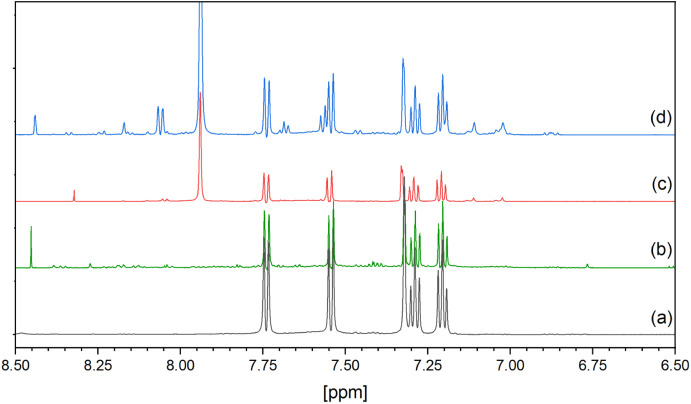
^1^H NMR spectra of (a) tryptophan; (b) tryptophan under UV light; (c) tryptophan with Ce-BA-DMF crystals treated in the dark; and (d) tryptophan with Ce-BA-DMF crystals under UV light. All spectra recorded in the H_2_O/D_2_O solution.

**Figure 8:**
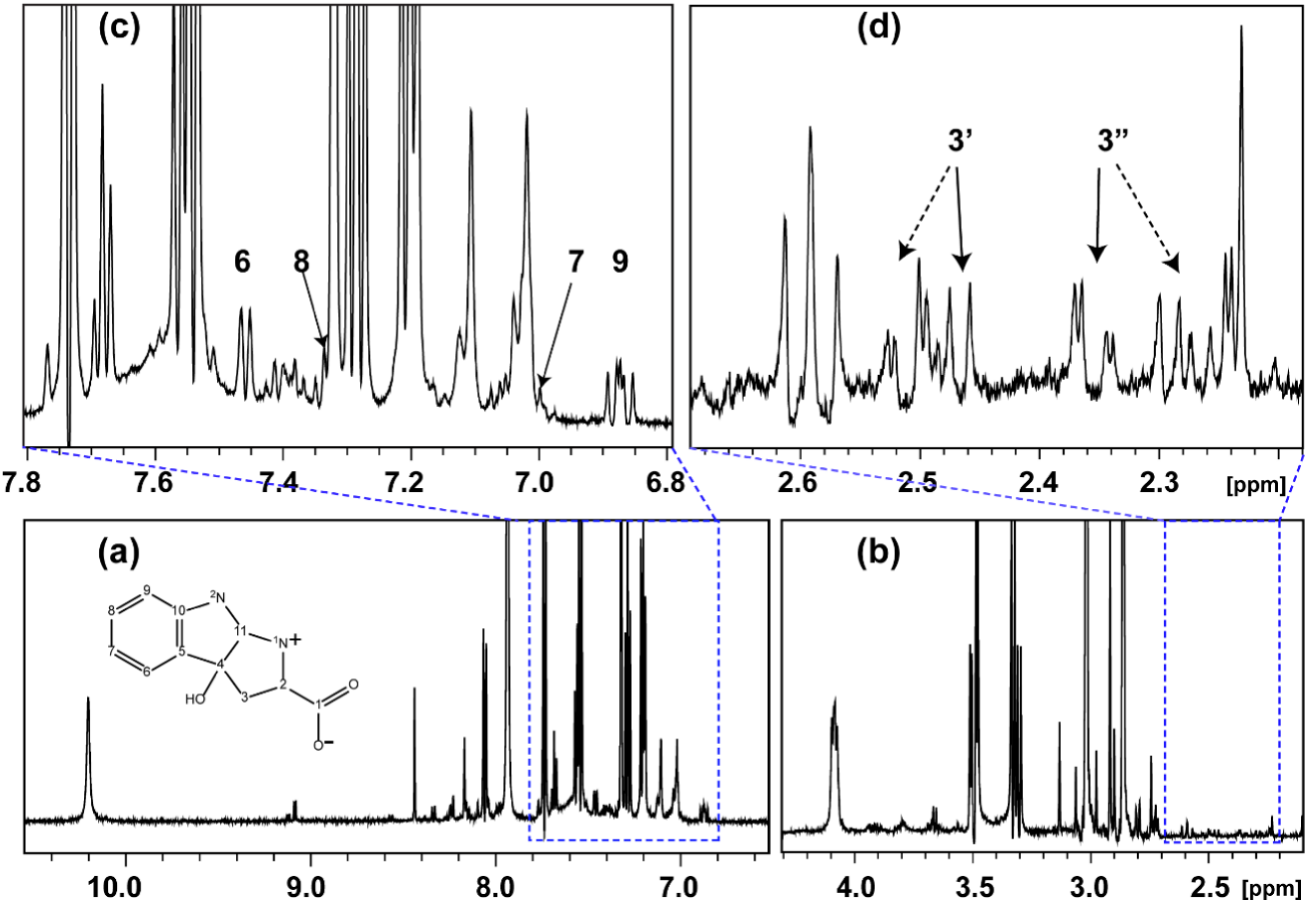
1D ^1^H spectrum of the aromatic and aliphatic regions of the Trp mixture with Ce-BA-DMF NPs is presented in panels (a) and (b), respectively. The expanded spectra arehighlighted in blue boxes and presented above panels (a) and (b) as the panels (c) and (d). Proton resonances belonging to the oxidized product of Trp are assigned. The structure of the oxidized product is shown in panel (a).

First, we conducted experiments in an aqueous solution. Ce-BA-DMF crystals were added to the solution containing tryptophan and kept either in the dark or under UV light for 7 days. Samples of the mother liquor were separated by sedimentation and analyzed by ^1^H NMR spectroscopy. The spectra of the tested samples, along with those of tryptophan kept in the dark and under UV light, are shown in Figures 7 and 8. In both tested samples, the proton resonances corresponding to the unaffected tryptophan were detected at 3.316 ppm (doublet of doublets), 3.494 ppm (doublet of doublets), 4.085 ppm (multiplet), 7.204 ppm (triplet), 7.287 ppm (triplet), 7.324 ppm (singlet), 7.541 ppm (doublet), and 7.736 ppm (doublet). Additionally, a few signals associated with DMF (2.863, 3.019, and 7.936 ppm, all singlets) and benzoic acid (7.559 and 7.684 ppm, triplets; 8.059 ppm, doublet) were observed, indicating the partial dissolution of the crystals in water. After UV radiation in the presence of Trp, we observed color changes in the Ce-BA-DMF crystals. The bulk material (crystals) changed color from yellow to brown, indicating the reduction of Ce(IV). The heterogeneous system can store electrons, which can be implied by the color changes.

According to Figure 7c, there is a slight change in the ^1^H NMR spectra of tryptophan treated with Ce-BA-DMF crystals in the dark. However, when tryptophan is exposed to UV light in the presence of Ce-BA-DMF nanoparticles, multiple new resonances appear in the spectrum, likely corresponding to oxidation products of tryptophan. Some new products may result from UV irradiation alone. It is well established^36^ that UV light induces a chain of oxidation products in tryptophan (Figure 7b).

Importantly, new resonances (approximately 6% relative to the parent tryptophan signal), not observed in the UV-only treatment appear in Figure 8a,b. A set of aromatic and aliphatic resonances in the ^1^H spectrum can be attributed to a tricyclic oxidation product of tryptophan, previously characterized by us^8^ in the reaction mixture of Trp with Ce(−)(C) NP. In that study, we showed that in this type of reaction, Ce(−)(C) nanoparticles convert tryptophan into a tricyclic organic acid belonging to the auxin family of natural plant hormones. Moreover, the conversion kinetics in the case of crystals tested in this paper are much higher compared to CeO, 6% per 7 days for Ce-BA-DMF crystals compared to 6% per 30 days for CeO(−).^8^

Figure 8c,d presents the assignment of resonances corresponding to this tricyclic oxidation product, along with its structure. Proton assignments were further confirmed by 2D HSQC and HMBC experiments, with their superpositions shown in Figure S6. In these figures, observed cross-peaks between ^1^H and ^13^C correspond to the expected structure of the oxidation products.

**Figure 9:**
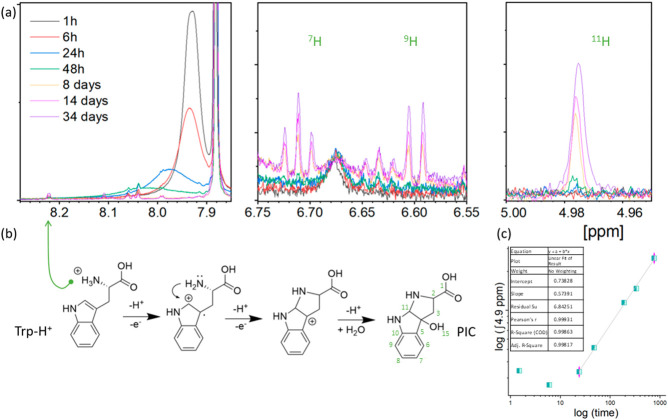
(a) ^1^H NMR spectra of tryptophan in the DMSO-d_6_ solution with Ce-BA-DMF crystals. (b) Scheme of the oxidation path and (c) kinetics of PIC concentration change.

The homogeneous investigation of the oxidation process of tryptophan molecules by Ce-BA-DMF crystals was done in DMSO-d_6_ solution (2.50 ppm). DMSO was chosen as a good solvent for Ce-BA-DMF crystals as well as Trp. The ^1^H NMR spectra were acquired during the period of 32 days (Figure 9a). The chemical shifts corresponding to benzoic acid and DMF from the crystal structures are clearly detected. Indeed, the methyl groups and formyl proton of DMF are observed at ca. 2.89 and 7.98 ppm, respectively. The aromatic protons from benzoic acid appear as multiplets between 7.2 and 8.2 ppm. The carboxyl proton, which is usually detected around 12 ppm, is not visible in the case of the tested crystals. During the tested period of oxidation, lines associated with Ce-BA-DMF molecules are visible, confirming the catalysis process rather than a chemical reaction between Trp and Ce-BA-DMF molecules (see Figure S7).

The ^1^H proton resonances associated with free tryptophan molecules are detected during the entire testing period, and their assignments are labeled in Figure 9. However, after 14 days, a number of new chemical proton resonances were observed, which can be attributed to the tryptophan oxidation product.

From our previous study,^8^ we predicted that the ^1^NH_2_ groups would be protonated, which resulted in the formation of the ammonium ion (see Scheme Figure 9b). This is evident from the appearance of a new resonance at 8.2 ppm (Figure 9a). Due to the simultaneous release of a proton and an electron, the indole unit cyclization is induced by the protonation of the amino group (see Scheme Figure 9b). The ^3^H of new appeared indole cyclic unit give a chemical shift at 2.64 and 2.66 ppm.^34^ Chemical shifts appearing at 4.98 ppm (singlet) correspond to the ^11^H (cyclic) and ^15^H protons (cyclic hydroxyl). Shifts associated with aromatic protons (^9^H and ^7^H) were detected at 6.60 ppm (doublet) and 6.71 ppm (triplet). As a result, the oxidation is a chemically specific process that leads to hydroxypyrroloindole carboxylic acid (PIC),^8,34^ as presented in Figure 9b.

The initial ^1^H NMR spectra show a single chemical shift corresponding to the Trp cyclic NH proton located at 10.7 ppm. This shift and the new chemical shift that appeared at 4.98 ppm associated with the indole cyclic proton (^11^H–PIC; see Figure 9a) were chosen for studying the kinetics of the oxidation process. The area under the initial NH shift was normalized to 100, and the chemical shift at 4.98 ppm associated with the new indole cyclic proton was normalized proportionally. The normalized area value represents the increase in PIC concentration. Figure 9c presents the PIC concentration changes as a function of time in a double logarithmic scale. The el, eqs 1 and 2.
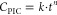
1
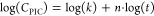
2

where *C*_PIC_ is the PIC concentration; *t* is time; *k* is constant; *n* is power law exponent. The results showed that the oxidation process requires a period of time to begin, approximately 24 h. Thereafter, the process continues, with the conversion rate around 0.1% per day. In case of the process of growing plants, slow and stable oxidation is a desirable feature of a given material.

The interaction between Ce-BA-DMF crystals and tryptophan was evaluated by measuring the concentration ratios of 1:1, 1:10, and 1:50 in MeOD. Depending on the ratios, the signals of aromatic tryptophan shift to the following: 7.72 to 7.46; 7.38 to 7.32; 7.21 to 7.10; 7.14 to 7.09; and 7.06 to 6.97 ppm (see Figures S8 and S9). The observed chemical shifts of the aromatic protons of tryptophan indicate that Trp is involved in a quick exchange between free and bound states with Ce-BA-DMF nanoparticles. This implies direct electron transfer between reducing and oxidizing agents.^37^ As a consequence of this slow exchange, the Ce-BA-DMF:Trp complex appears in the solution.

It is important to note that Ce-BA-DMF, as an oxidation catalyst, can easily be applied in biological systems for the selective oxidation of amino acids and proteins, exploiting the well-established approach based on the addition of a water-insoluble active agent in a DMSO solution to a water solution of the substrate to be treated, in the same way as water-insoluble drugs are ordinated for local delivery, for example, for penetration via the skin.^38^ DLS studies have clearly indicated that the complex is stable and does not aggregate in solutions containing up to 30% of water by volume. It has also apparent advantages compared to other reported Ce-based molecular catalysts. In our tests, we compared the new catalyst to Ce^4+^ ions in solution and observed that the latter (applied as Ce(SO_4_)_2_ solution with the same concentration with respect to Ce) quickly resulted in the complete oxidation of Trp, with no proton CH signals observable in NMR. Another potential Ce-based oxidation catalyst for biological applications, the Keggin sandwich [Ce(IV)(PW_11_O_39_)_2_]^10–^, was apparently slowly reduced in the presence of amino acids, but no oxidation products could be detected by NMR except for cysteine. It was even suggested that Trp was restored in the reaction with this catalyst, making it not suitable for its oxidation.^39^

## 3 Conclusions

Here, a new ceria oxo-complex has been successfully synthesized and characterized. This crystal phase was found to be stable after drying and when left for 24 h in an aqueous solution up to pH 10. The obtained crystals exhibited low solubility in the tested pH range of 4–10. The catalytic properties of Ce-BA-DMF in the oxidation of ABTS and tryptophan were also investigated, where it was possible to detect the products of oxidation for both molecules. It was demonstrated by cyclic voltammetry measurements that Ce-BA-DMF had a Ce(III)/Ce(IV) couple positive enough to directly oxidize Trp via subsequent steps with the release of a proton and an electron. In this way, an amino group is protonated, and a new indole cyclic unit is formed. The conversion kinetics of the crystals examined in this paper are significantly quicker than those of CeO_2_ NPs. An in-depth study of molecular interaction kinetics was conducted in nonaqueous media. The oxidation kinetics fit a power-law model, with the conversion speed being relevant for potential applications in plant growth stimulation. The successful synthesis resulted in the formation of molecular species representing the surface of ceria oxide NPs, and a direct molecular mechanism is proposed.

## 4 Materials and Methods

### 4.1 Materials

The synthesis substrates and chemicals used for the investigation were of analytical grade. The crystals of Ce-BA-DMF were obtained with the starting substrates (NH_4_)_2_Ce(NO_3_)_6_ (≥98.5%, Sigma-Aldrich), benzoic acid C_6_H_5_COOH (pure, pharma grade, PanReac AppliChem), and dimethylformamide (DMF) HCON(CH_3_)_2_ (≥99.8%, Sigma-Aldrich). The NMR tests were done with D_2_O (99.97%, Euroisotop); deuterated dimethyl sulfoxide (DMSO-d_6_) (CD_3_)_2_SO (99.9%, Cambridge Isotope Laboratories); and deuterated methanol (MeOD) CD_3_OD (99.96%, Euroisotop). The oxidation tests were performed in the presence of l-tryptophan (≥98%, Sigma-Aldrich) and 2,2′-azino-bis(3-ethylbenzothiazoline-6-sulfonic acid) diammonium salt (ABTS) (≥98%, Sigma-Aldrich). For pH stability and solubility studies, diluted solutions of HCl (≥37%, Sigma-Aldrich) and NH_4_OH (25%, AnalaR) were used.

### 4.2 Synthesis

Crystals were obtained from the solution. First, 0.0732 g of benzoic acid was dissolved in 1.8 mL of DMF or acetonitrile (MeCN). Then, 0.6 mL of (NH_4_)_2_Ce(NO_3_)_6_ aqueous solution (0.5 M) was added. The mixture was stirred for 24 h, and then the solution was left for slow evaporation at room temperature. After 7 days (Ce-BA-DMF) and 28 days (Ce-BA-MeCN), well-defined, yellow crystals were found.

### 4.3 Single-Crystal X-ray Diffraction

To collect the single-crystal X-ray diffraction data, a Bruker D8 SMART APEX II CCD diffractometer (operating with graphite-monochromated Mo–Kα radiation, λ = 0.71073 Å) was used. Data were collected at room temperature. For details of data collection and refinement, please see Supporting Information.

### 4.4 Electrochemical Measurements

The studies were carried out using a Metrohm Autolab PGSTAT204 potentiostat. All electrochemistry was performed in a single-chamber cell using a glassy carbon electrode (*A* = 0.071 cm^2^) (Redoxme AB) as the working electrode, a platinum wire as the counter electrode. In organic electrolyte (100 mM TBA PF_6_ in MeCN solvent), an Ag/AgNO_3_ electrode was used as a pseudoreference, with the ferrocene/ferrocenium redox couple used as an internal reference. In aqueous electrolyte (100 mM KCl), an Ag/AgCl reference was used. Values were converted to NHE using the reported potentials of the ferrocene/ferrocenium couple (+0.630 V_NHE_) and the Ag/AgCl electrode (+0.197 V_NHE_) vs NHE.^40,41^ Cells were purged with N_2_ for 10–15 min prior to experiments. All electrochemistry was performed at room temperature and pressure.

### 4.5 NMR

The NMR data were recorded on Bruker Avance III spectrometers, operating at 14.1 T, equipped with a cryo-enhanced QCI-P probe at a temperature of 298 K. For the assignment of the chemical shifts of the oxidized Trp product, Bruker standard pulse sequences of 2D TOCSY, HSQC, HMBC, and NOESY were used. Spectra were processed with TopSpin 4.3.0. All spectra were acquired in 5 mm NMR tubes (final volume of 0.500 mL). For experiments in water, all spectra were referenced to an external ^1^H chemical shift standard, 0.1 mM DSS (4,4-dimethyl-4-silapentane-1-sulfonic acid), and ^13^C chemical shifts were referenced indirectly to the ^1^H standard using a conversion factor derived from the ratio of NMR frequencies.

To experiment in DMSO and methanol, the solvent signals were used for referencing the proton spectra.

Experiment 1–0.060 g of Ce-BA-DMF crystals were placed into 5 mL of tryptophan aqueous solution (*C_P_* = 5.0 mg/mL). One sample was covered with aluminum foil and left stirred. The other test was performed under a UV lamp (Osram Ultra-Vitalux 300W Simulated Sunlight) with stirring. After 7 days, the solutions were filtered and centrifuged. Finally, 225 μL of the sample, together with 25 μL D_2_O were placed in an NMR tube.

Experiment 2–For the second experiment, DMSO was used as the solvent. The molar ratio of Ce-BA-DMF crystals to tryptophan was set at 1:1 and dissolved in DMSO-d_6_ NMR solvent. The solution was placed into an NMR tube, and the experiment was run after 1, 4, 25, and 48 h and 7, 14, and 32 days.

Experiment 3–In the 5.0 mM solution of tryptophan in MeOD, the Ce-BA-DMF crystals (5 mmol) were dissolved. Final molar ratio of tryptophan molecules to Ce-BA-DMF crystals were equal to 1:1, 1:10, and 1:50 molar. The NMR spectra were recorded immediately. To compare, tryptophan and Ce-BA-DMF crystals were also dissolved separately in MeOD, and NMR spectra were collected.

### 4.6 Scanning Electron Microscopy

The Flex-SEM 1000 scanning electron microscope, combined with the AZtecOneXplore EDS detector by Oxford Instruments (UK) and energy dispersion spectroscopy (EDS) from Hitachi (Tokyo, Japan), was used to record SEM images for morphology investigation and EDS measurements and mapping. The images were detected under an acceleration voltage of 20 kV, a spot size of 50, and a working distance of 10 mm. Elemental content was checked on the surface of 5 different crystals.

### 4.7 pH Stability

The stability of the crystal structure was tested in different pH environments. For this, HCl solutions with pH of 4 and 5 (concentration of HCl: 10^–5^ M), water with a pH of 5, and NH_4_OH with pH in the range of 6–10 were tested. The known amount of Ce_BA_DMF crystals (≈0.01 g) was placed into the solution (5 mL) and stirred for 24 or 48 h. After the set time, the crystals were removed, placed in oil, and tested by XRD to determine their structure.

### 4.8 Kinetic of Catalysis

The oxidation of ABTS was monitored as the changes in absorption in the wavelength range of 250–900 nm. Tests were conducted with a mixture of water and ethanol or acetonitrile in a volume ratio of 1:1. Crystals and ABTS were mixed in both solvent systems at molar ratios of 2:1, 1:1, and 1:2. Absorption was measured by a UV–Vis spectrometer, Multiskan Sky High (Thermo Fisher Scientific, Waltham, MA, USA). A solution of Ce-BA-DMF crystals and ABTS was placed in a standard 96-well plate.

### 4.9 FTIR

The spectra were recorded in transmission mode using samples ground with dried KBr as a matrix, pressed into pellets 1 cm in diameter and ca. 0.5 mm thick. A PerkinElmer Spectrum 100 instrument was used for registering the spectra in the 4000–400 cm^–1^ range, with a scan step of 4 cm^–1^ and 8 scans per spectrum.

### 4.10 DLS

Dynamic Light Scattering experiments were carried out with the Malvern Panalytical Zetasizer Ultra instrument. Ce-BA-DMF was dissolved in pure DMSO (up to 10 mM concentration), then filtered H_2_O was added dropwise to reach ∼30% before any noticeable precipitation occurred. The sample was clarified by centrifugation and run in DLS for ∼ 60 min at 25 °C with no appreciable change in radius.

## Supporting Information Available

Additional details on the synthesis of applied model substances (DOC), crystal and molecular structure of the Ce-BA-AN compound (Figure S1); structure solution and refinement details for the Ce-BA-AN compound (Table S1); solubility of Ce-BA-DMF crystals as a function of solution pH (Figure S2); CVs (a) at varying scan rates of Ce-BA-DMF in MeCN (Figure S3); experimental details on the ABTS oxidation (DOC and Figures S4 and S5, showing kinetic data and the recorded spectra, respectively); details of the NMR experiments (DOC) and 2D NMR spectra with assignment of the oxidized product of Trp obtained in a mixture with Ce-BA-DMF (Figure S6); ^1^H NMR spectra from the first oxidation experiment, performed in a water solution of tryptophan in the presence of not soluble Ce-BA-DMF crystals (Figure S7); ^1^H NMR spectra of tryptophan kept with Ce-BA-DMF crystals in the MeOD solvent (Figure S8); ^1^H NMR spectra of tryptophan with Ce-BA-DMF crystals in the MeOD solvent (Figure S9); FTIR spectra of fresh single crystals of Ce-BA-DMF (A), Ce-BA-DMF powder after 1 week of exposure to Trp solution in darkness (B), Ce-BA-DMF powder after 1 week of exposure to Trp solution under UV (375 nm), and a comparison of all three spectra in the 400–700 cm^–1^ region characteristic of M-O bond vibrations (Figure S10); DLS of the Ce-BA-DMF in water-DMSO solution (Figure S11) (PDF)
