# Supplementary material for: Visualizing Catalytic Oxidation of Tryptophan by Nanoceria via an Oligonuclear Cerium Oxo-Complex Model
Source: Inorg Chem. 2025 Apr 10;64(15):7300–10. doi: 10.1021/acs.inorgchem.4c05165 (PMC12015816; doi:10.1021/acs.inorgchem.4c05165)
Supplement: Supplementary file 1 — ic4c05165_si_001.pdf [file ic4c05165_si_001.pdf]

SUPPORTING INFORMATION

Visualizing catalytic oxidation of Tryptophan by nanoceria via oligonuclear cerium oxo-complex model

SARA TARGONSKA[1], FRANCESCA GREENWELL[2], TATIANA AGBACK[1], GULAIM A. SEISENBAEVA[1], AND VADIM G. KESSLER[1,]*

[1] DEPARTMENT OF MOLECULAR SCIENCES, SWEDISH UNIVERSITY OF AGRICULTURAL SCIENCES, BOX 7015, 750 07 UPPSALA, SWEDEN,

Vadim.Kessler@slu.se

[2] DEPARTMENT OF CHEMISTRY, UPPSALA UNIVERSITY, BOX 523, 751 20 UPPSALA, SWEDEN

1. MATERIALS AND METHODS

1.1. MATERIALS

The synthesis substrates and chemicals used for the investigation were analytical grade. For the synthesis were applied: benzoic acid (PanReac AppliChem, cas no: 65-85-0), acetylsalicylic acid (Duchefa Biochemie cas no: 50-78-2), salicylic acid (Duchefa Biochemie, cas no: 69-72-7), and p-aminobenzoic acid (Duchefa Biochemie, cas no: 150-13-0). As solvents for synthesis were used: Dimethylformamide (DMF) $HCON(CH_3)_2$ (≥99.8%, Sigma Aldrich), and acetonitrile (99.7%, Sigma Aldrich). For all trials the $(NH_4)_2Ce(NO_3)_6$ (Sigma Aldrich) were used as cerium ions source.

1.2. SYNTHESIS

All tested were performed with the same procedure. Stoichiometric amount of organic substrate was dissolved with 1.8 mL DMF, or acetonitrile (MeCN), and water solution of

$(NH_4)_2Ce(NO_3)_6$ was added (V=0.6 mL C_M=0.5 M). After stirring for 24h, solutions were left for slow evaporation.

2. RESULTS

2.1. Ce-BA-MeCN crystal structure

By the chosen synthesis path the new crystal structures were obtained. Aside of the structure Ce-BA-DMF presented in the main text, the Ce-BA-MeCN was obtained. A well-defined matrix of yellow cubic/rhombohedral crystals appeared at the bottom of the vessel after approximately 4 weeks of incubation. In order to investigate the crystals structure, single X-ray diffraction was used. Same as in case of Ce-BA-DMF crystals, the core is built with 6 Ce atoms connected with 8 oxygen. This core is surrounded by three nitrate groups and nine benzoate ligands, each bridging to the core via both oxygen atoms. Additionally, three protonated benzoic acid molecules are bonded to the ceria-oxygen core through only one oxygen atom. The chemical formula of composition is $Ce_6O_4(OH)_4(H_2O)_2(NO_3)_3(C_7H_5O_2)_9(C_7H_6O_2)_3$, with the molar mass equal to 2935.75 g/mol, synthesis yield 31%, and the size on one molecule 19.033 Å. On the **Figure S1** the representative of one molecule and the packing are depicted. The bond between Ce01 and nitrile group is equal to 2.760 Å. Between all three Ce01 atoms, there is one oxygen, and the one water molecule with the hydrogen bond equal to 2.811 Å. The unit cell parameters and the details of data collection are listed in **Table S1**.

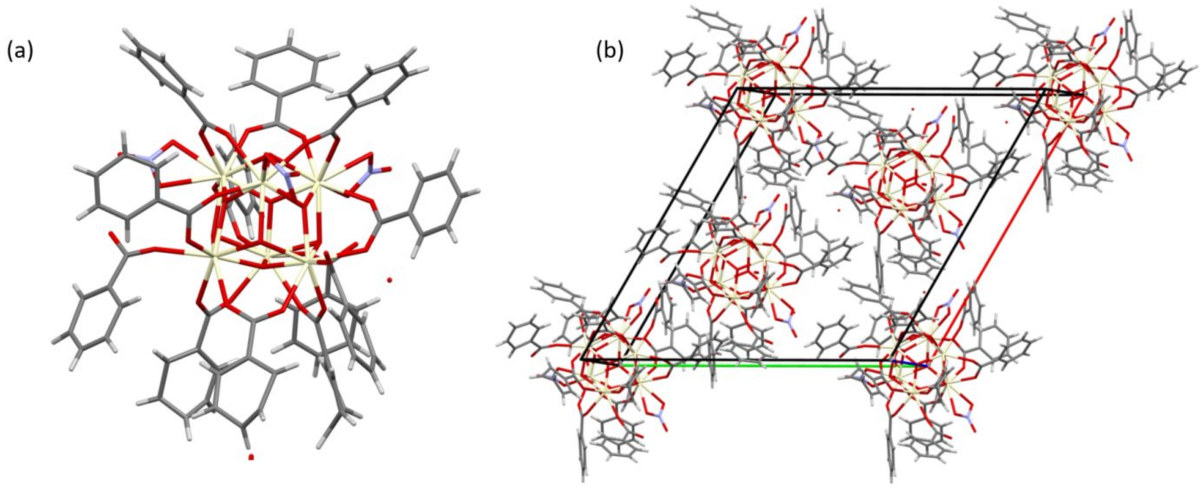

Figure S1. (a) Crystal structure of Ce-BA-MeCN molecule, (b) packing of Ce-BA-MeCN unit cell; Color scheme: Ce - green, O - red, N = blue, C = gray

Table S1. Details of unit cell parameters and data collection of Ce-BA-MeCN crystals.

Compound	Ce-BA-MeCN
**Crystal system**	Rhombohedral
**Space Group**	R-3

**Space group number**	146
**a, b [Å]**	23.9536(11)
**c [Å]**	16.0273(10)
**V [Å³]**	7964.0(9)
**α, β [°]**	90.0
**γ [°]**	120.0
**T [K]**	296(2)
**Z**	6
**Nr. of obs. independent refl., I > 2σ(I)**	9847

2.2. Solubility

The concentration of cerium after incubation in the solution of different pH for 48h was measured by ICP-OES. Base on then raw data showing the Ce content the solubility of crystals was calculated. The final solubility of the Ce-BA-DMF crystals in the various pH solutions is depicted on **Figure S2**.

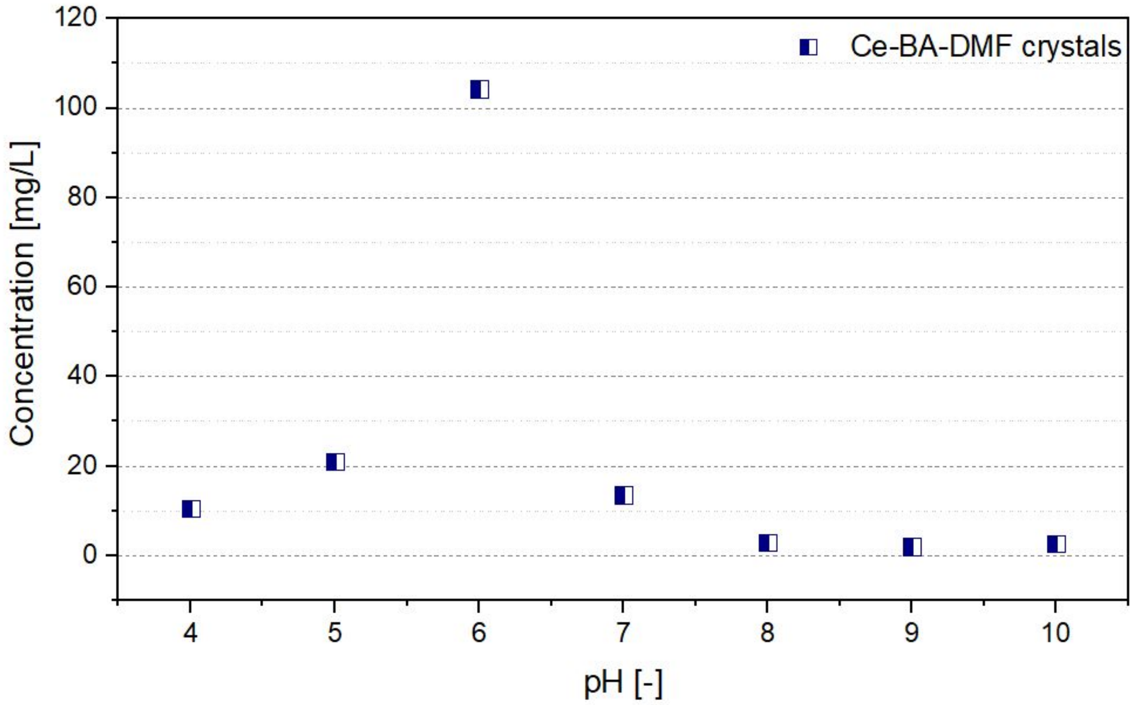

Figure S2. Solubility of Ce-BA-DMF crystals as a function of solutions pH

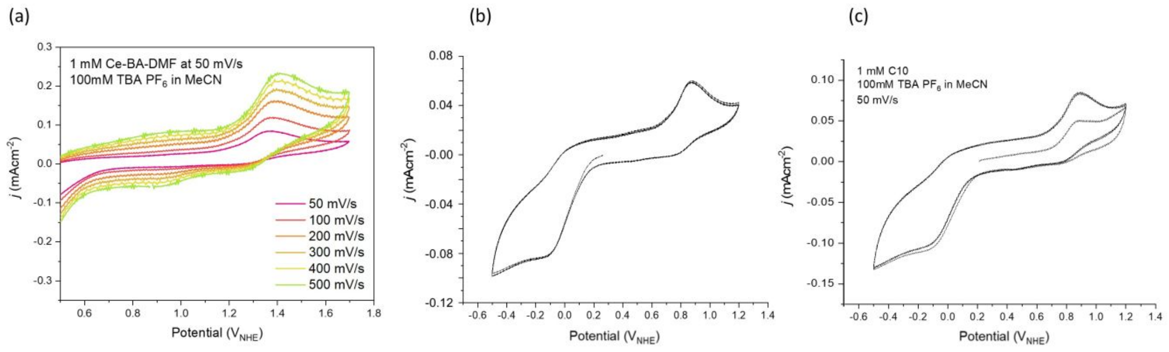

Figure S3. CVs (a) at varying scan rate of Ce-BA-DMF in MeCN; (b) Starting from OCP scanning negative; (c) Starting from OCP scanning positive

2.3. ABTS oxidation

The graphs in **Figure 6** and **S4** illustrate the changes in spectra from 250 to 900 nm over a period of 4 hours, recording the process of ABTS oxidation. The integration area of the wavelength in range of 340–350 nm and 415-425 nm associates with concentration of non-oxidized ABTS and $ABTS^{\bullet+}$ products, respectively. [1–3] It has been noted that the absorption of light at higher energies (centred at 280-310 nm) is a result of Ce(IV) ions, if it presents in the solution. However the reduced form of ABTS has absorption in close wavelength range (290-370 nm) (see spectra's deconvolution **Figure S5**.). Since the absorption spectra overlap, some errors may occur in the analysis of changes in the concentrations of ABTS.

The ethanol-water mixture used as a solvent the ABTS oxidation kinetic (see **Figure S4**) is slightly different comparing to water:MeCN (**Figure 6**). Regardless of tested ratio between ABTS and oxidant agent, the oxidation rate is equal over 80 minutes of monitoring reaction with following up increasing rate if excess of ABTS is measured at ratio 1:2 of Ce-BA-DMF:ABTS. The equal molar ratio and excess of Ce-BA-DMF oxo-complex results in decreasing of $ABTS^{\bullet+}$ cation concentration. At the same time concentration of reduced form of ABTS, monitored at 340 nm evenly decreases. There is a drop in the level of 8%, 15%, and 32% in the case of equal molar ratios, crystal excess, and ABTS excess, respectively. About 15% of the ABTS concentration is reduced by non-promoted oxidation in the mixture of EtOH:H_2O solution.

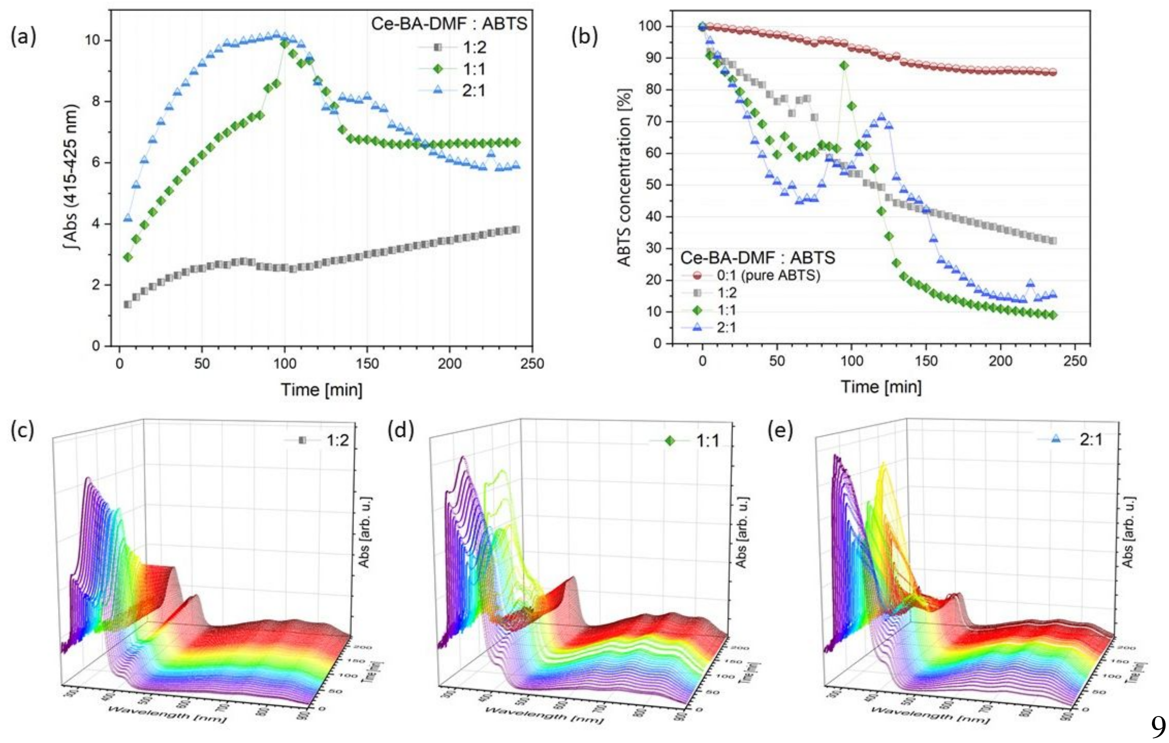

Figure S4. The oxidation process of ABTS in the ethanol solution (a) the mathematical area under UV-Vis absorption spectra in the range of 415-425 nm (b) non-oxidized ABTS concentration (c) spectra recorded in the ratio of crystals:ABTS 1:2; (d) in the ratio of crystals:ABTS 1:1; (e) in the ratio of crystals:ABTS 2:1

Deconvolution of absorption spectra of the ABTS oxidation in the presence of Ce-BA-DMF crystals is shown on the **Figure S5**. The deconvolution was done for the spectra recorded after 5 minutes and 235 minutes of oxidation.

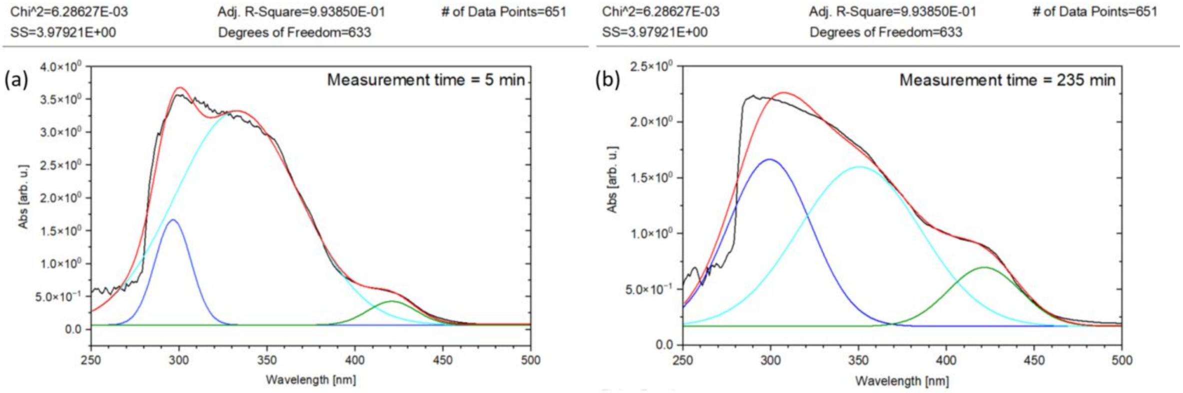

Figure S5. Deconvolution of the absorption spectra of ABTS oxidation in acetonitrile:water solution under presence of Ce-BA-DMF, molar ratio 2:1 (Ce-BA-DMF:ABTS) recorded after (a) 5 min, and (b) 235 min

2.4. NMR measurements

The **Figure S6** presents the 2D NMR spectra of with assignment of the oxidized product of Trp obtained in a mixture with Ce-BA-DMF.

The **Figure S7** shows the ^{1}H NMR spectra of first oxidation experiment, performed in water solution of tryptophan in the presence of not soluble Ce-BA-DMF crystals. The spectra shows samples after 7 days in dark, and under UV lamp.

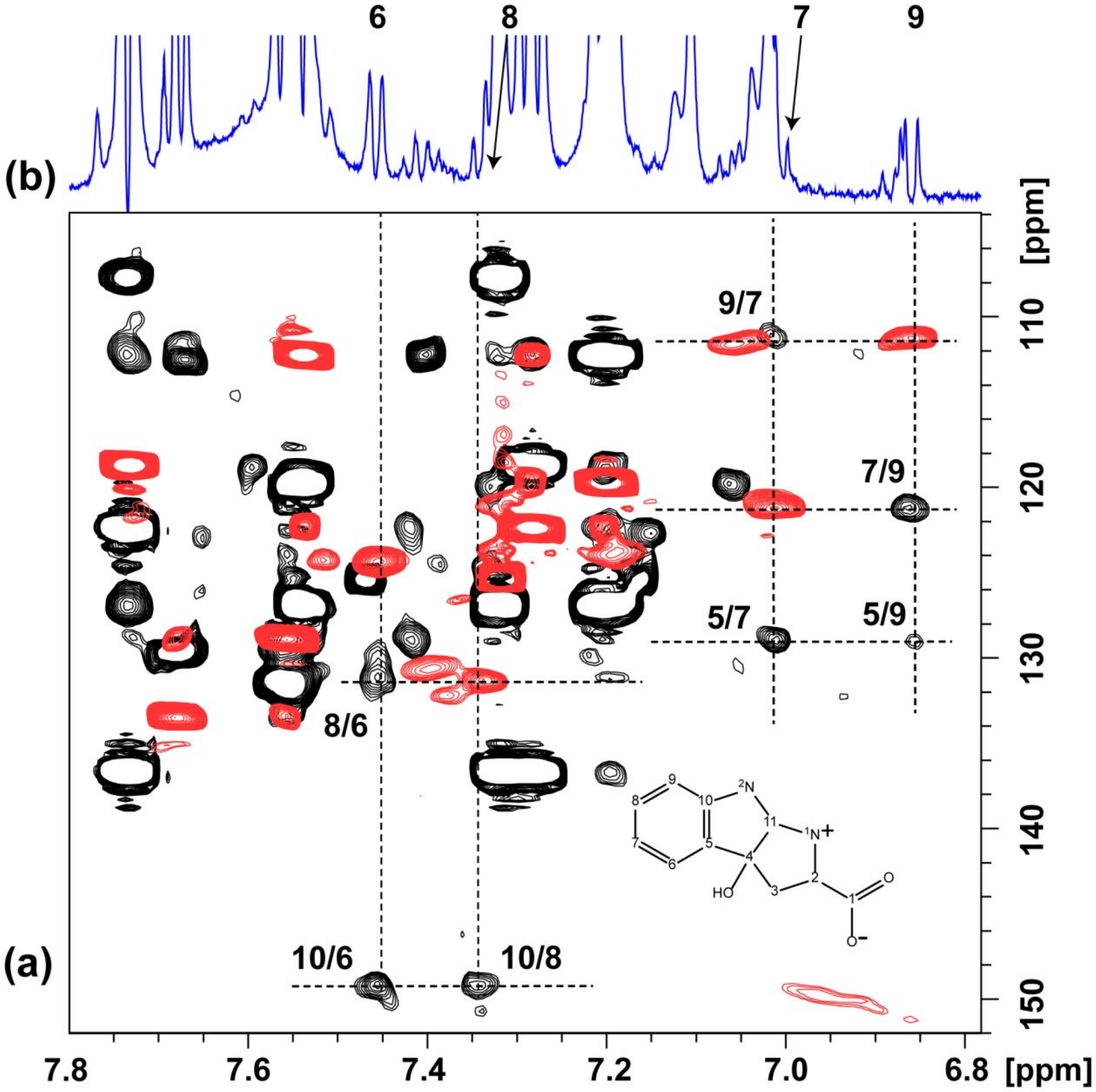

Figure S6. NMR spectra of Trp oxidation products. The superposition of the HMBC (black) and HSQC (red) spectra is presented in the aromatic 7.8-6.8ppm (a) region. The ^{1}H spectrum of the same region is shown in (b). Assignment and numbering are as indicated according to the structure in panel (a).

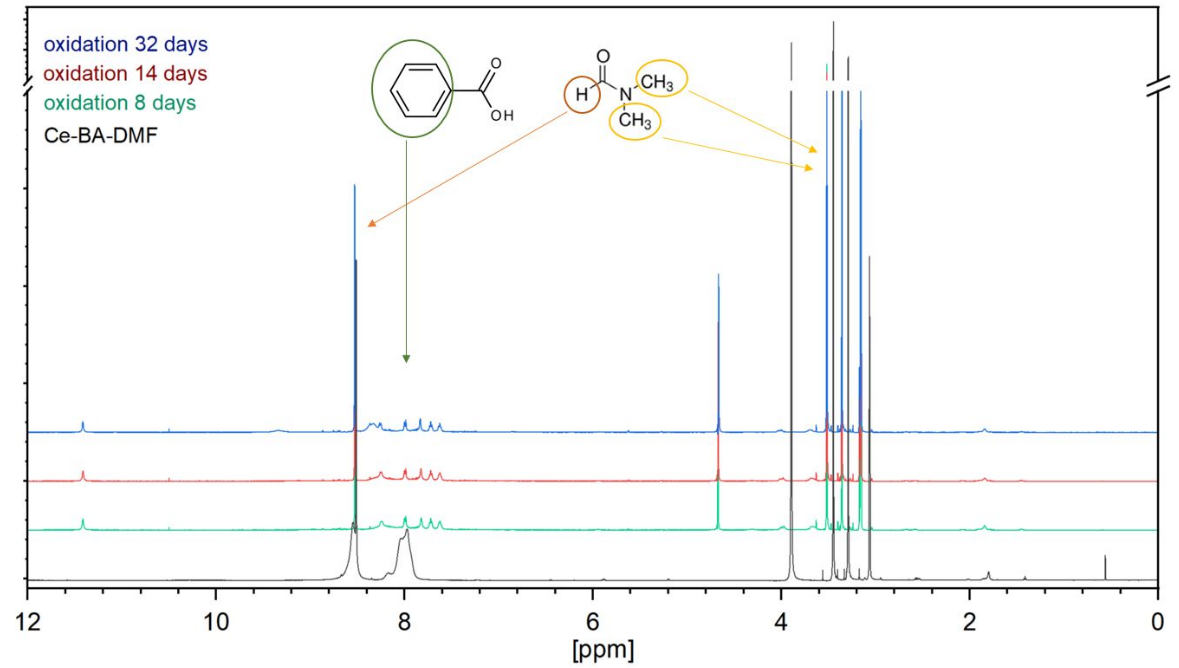

Figure S7. NMR spectra of pure Ce-BA-DMF crystals and oxidation of Trp after 8, 14 and 32 days, DMSO-d6

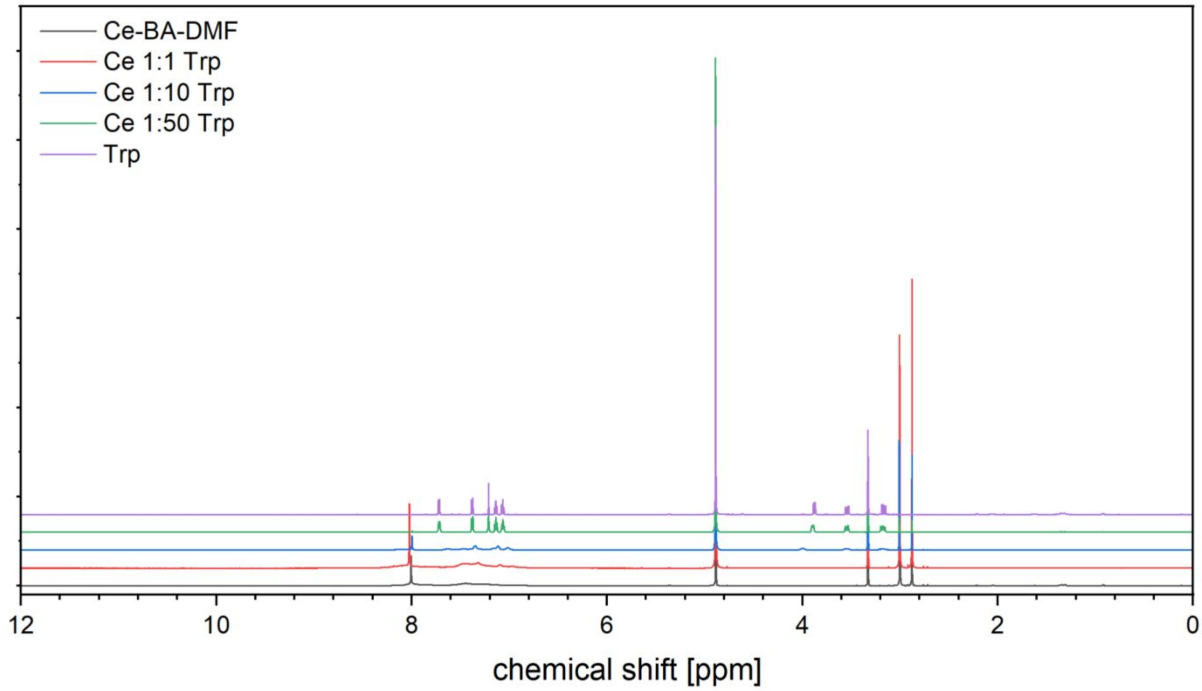

Figure S8. ^{1}H NMR spectra of the tryptophan with Ce-BA-DMF crystals in the MeOD solvent

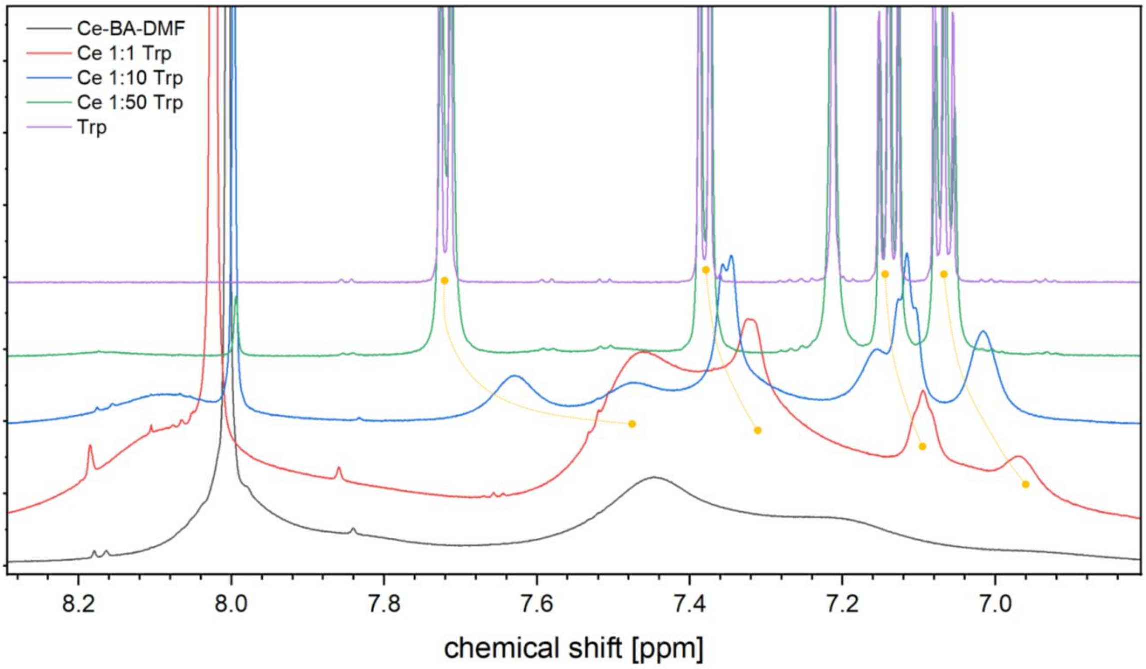

Figure S9. ^{1}H NMR spectra of the tryptophan with Ce-BA-DMF crystals in the MeOD solvent

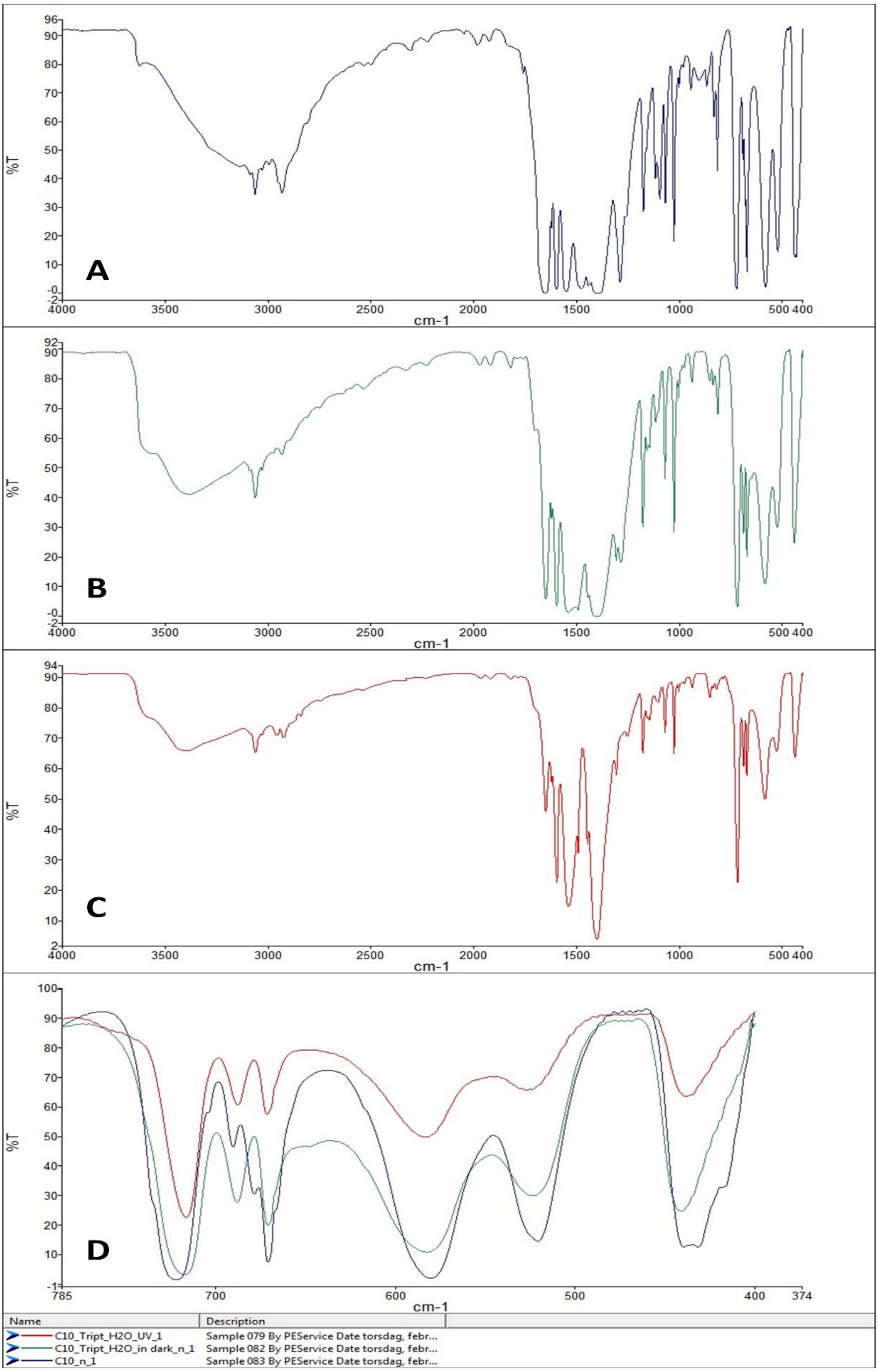

Figure S10. FTIR spectra of fresh single crystals of Ce-BA-DMF (A), Ce-BA-DMF powder from 1 week exposure to Trp solution in darkness (B), Ce-BA-DMF powder from 1 week exposure to Trp solution under UV (375 nm) (C), and comparison of all three spectra in the 400-700 cm-1 region characteristic of M-O bond vibrations (D).

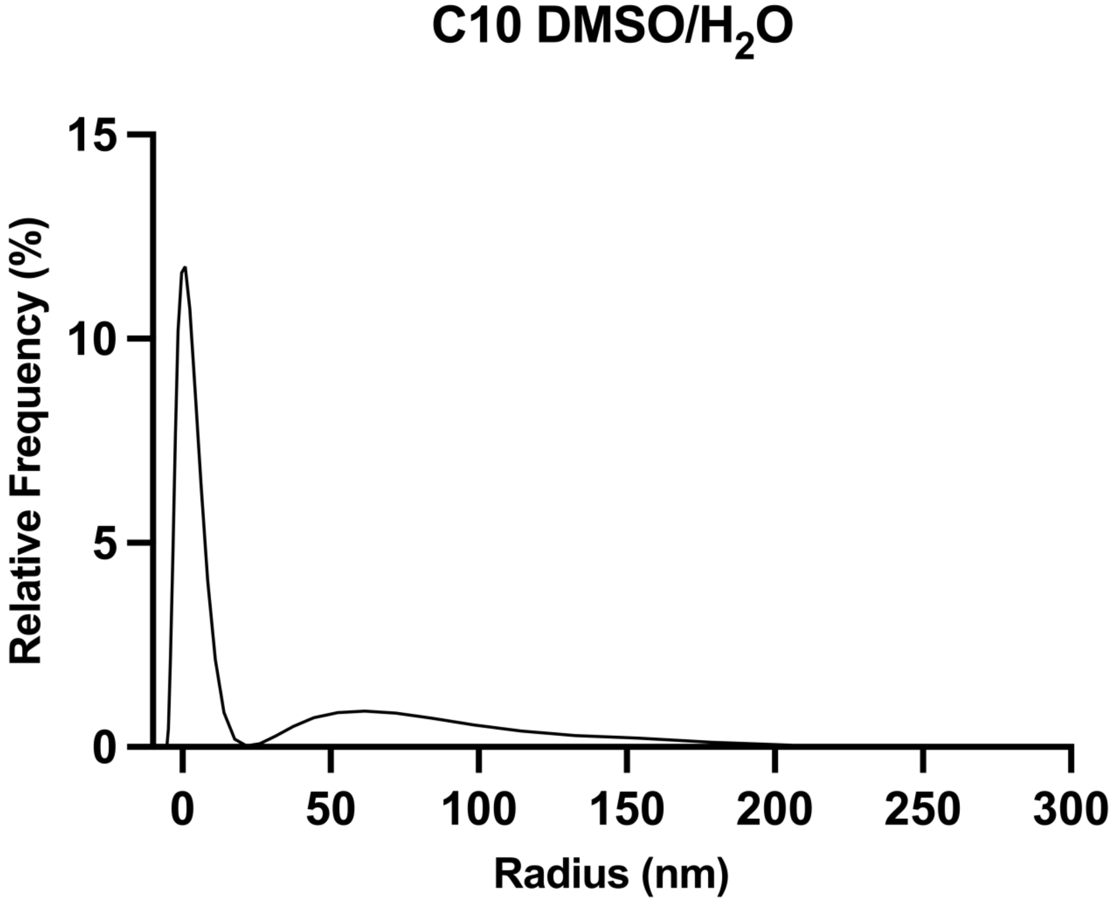

Figure S11. DLS analysis of hydrodynamic radius for Ce-Ba-DMF in 30% water in DMSO solution.

References

(1) Branchi, B.; Galli, C.; Gentili, P. Kinetics of Oxidation of Benzyl Alcohols by the Dication and Radical Cation of ABTS. Comparison with Laccase–ABTS Oxidations: An Apparent Paradox. *Org. Biomol. Chem.* **2005**, *3* (14), 2604–2614. https://doi.org/10.1039/B504199F.

(2) Guo, J.; Yang, L.; Gao, Z.; Zhao, C.; Mei, Y.; Song, Y.-Y. Insight of MOF Environment-Dependent Enzyme Activity via MOFs-in-Nanochannels Configuration. *ACS Catal.* **2020**, *10* (10), 5949–5958. https://doi.org/10.1021/acscatal.0c00591.

(3) Liu, H.; Zhou, P.; Wu, X.; Sun, J.; Chen, S. Radical Scavenging by Acetone: A New Perspective to Understand Laccase/ABTS Inactivation and to Recover Redox Mediator. *Molecules* **2015**, *20* (11), 19907–19913. https://doi.org/10.3390/molecules201119672.
